# Fever of Undetermined Origin During Neoadjuvant Chemoradiotherapy of Gastroesophageal Junction Adenocarcinoma Due to Radiation-induced Liver Disease

**DOI:** 10.7759/cureus.5803

**Published:** 2019-09-30

**Authors:** Babar Bashir, Andrew Song, Voichita Bar-Ad, Atrayee Basu Mallick

**Affiliations:** 1 Medical Oncology, Thomas Jefferson University Hospital, Philadelphia, USA; 2 Radiation Oncology, Thomas Jefferson University Hospital, Philadelphia, USA

**Keywords:** fever, fever of unknown origin, pet scan, gastroesophageal junction tumor, esophageal cancer, radiation-induced liver disease

## Abstract

Fever is a common occurrence in cancer patients and often attributed to the disease or treatment complications. Here we present a case of prolonged fever of undetermined origin in a patient undergoing neoadjuvant chemoradiotherapy for gastroesophageal junction (GEJ) adenocarcinoma. An initial detailed work-up remained elusive to the cause of the fever. However, a positron emission tomography (PET) scan prior to the planned surgical procedure identified a lesion in the liver concerning for hepatic metastasis. A prompt biopsy was obtained and revealed radiation-induced liver disease (RILD). Following treatment with corticosteroids, patient’s fever completely subsided. While RILD is a recognized complication of radiotherapy, prolonged fever in this setting has not been previously reported. This case illustrates that RILD can sometimes present as prolonged fever. Furthermore, clinicians should be cognizant of radiation necrosis as a potential treatment complication that should be confirmed with a biopsy to avoid missing the chance at potential cure.

## Introduction

Fever of undetermined origin (FUO) is defined as a fever that lasts more than three weeks duration with several temperature readings above 101°F and inability to establish diagnosis despite inpatient investigation [1]. Fever is common in cancer patients and a dreaded complication in the presence of neutropenia. In addition, 15%-20% of cancer patients develop tumor fever, a diagnosis of exclusion [2]. We present a rare case of prolonged fever without neutropenia in a patient receiving neoadjuvant chemoradiotherapy for gastroesophageal junction (GEJ) tumor attributed to radiation-induced liver disease (RILD).

## Case presentation

**Figure 1:**
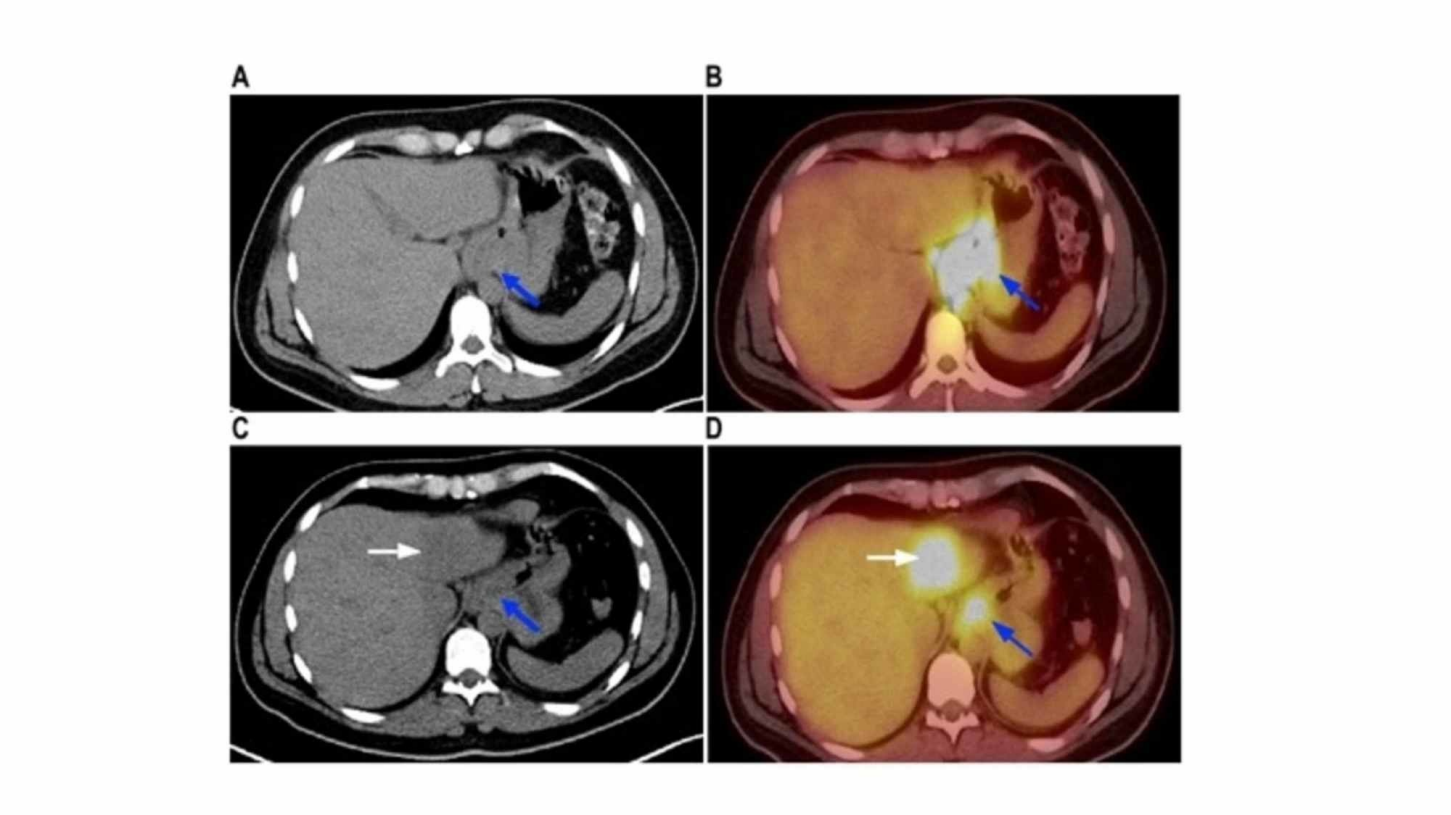
Pre- and post-treatment PET/CT scans. Pretreatment positron emission tomography/computed tomography (PET/CT) scan (panels A and B) show gastroesophageal junction (GEJ) mass and hypermetabolic lesion (blue arrow). PET/CT after completion of neoadjuvant treatment show a faint line of demarcation and hypermetabolic lesion in the left hepatic lobe (white arrow, panels C and D). A decrease in the hypermetabolic activity of the GEJ tumor is noted (blue arrow, panel D).

A 41-year-old Caucasian male with a history of gastroesophageal reflux disease presented with persistent and worsening dyspepsia. There was no prior history of allergies, autoimmune disease, or liver disease. Initial work-up with an esophagogastroduodenoscopy revealed a friable mass at the GEJ. Further investigation with endoscopic ultrasound and positron emission tomography/computed tomography (PET/CT) scan showed the mass to be 4 cm in size with paraesophageal and splenic axis lymphadenopathy (LAD) (Figure 1 A,B).

**Figure 2:**
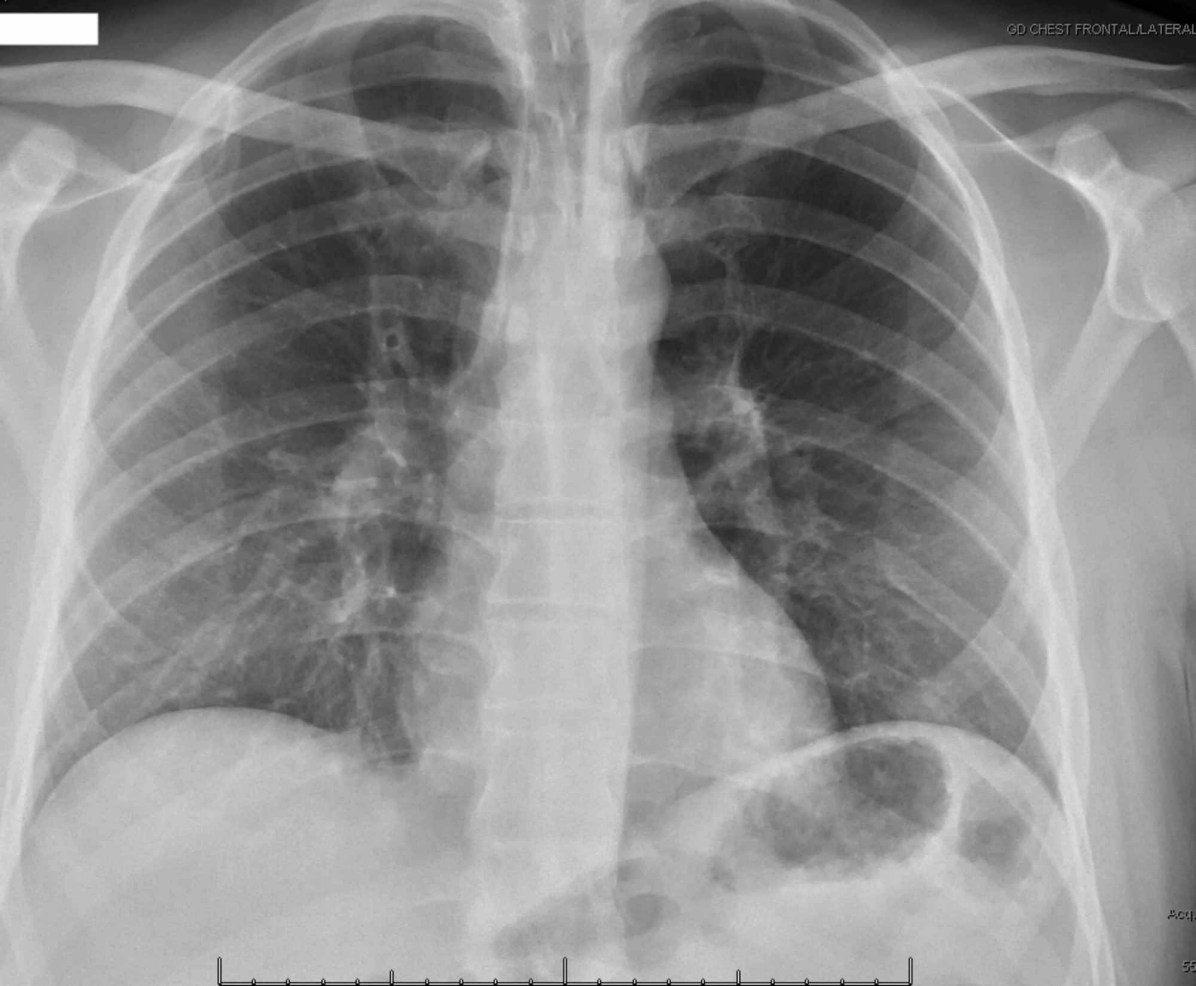
Normal chest X-ray. A chest X-ray revealed no significant abnormalities during fever work-up.

Pathology showed poorly differentiated invasive adenocarcinoma with clinical stage III (cT3N1M0) disease. He was started on neoadjuvant chemoradiotherapy with carboplatin, paclitaxel without port placement, and total radiation dose of 50.4 Gy in 28 fractions similar to the CROSS regimen with more traditional radiation dose [3]. Patient developed intermittent low-grade fever (<100.4°F or <38°C) without neutropenia one week after initiation of therapy. Initial infectious work-up including chest X-ray, blood, and urine cultures was unrevealing (Figure 2).

Subsequently he developed an episode of high-grade fever prompting ER evaluation where he was diagnosed with influenza A and treated. A week later, the low-grade fever returned and persisted throughout the course of neoadjuvant chemoradiotherapy despite using ibuprofen. A week after finishing the course of neoadjuvant chemoradiotherapy, however, his fever changed from intermittent low-grade to persistent high-grade (>101°F or >38.3°C) requiring hospitalization. A complete physical examination was unrevealing. Repeat cultures, echocardiography, interferon gamma release assay, legionella and streptococcal pneumoniae antigens, influenza A/B, 18-organism respiratory pathogen ribonucleic acid panel, and liver function panel (including bilirubin, aspartate aminotransferase, alanine aminotransferase, and alkaline phosphatase) were negative. A CT of the chest, abdomen, and pelvis was obtained to rule out metastasis causing tumor fever or occult abscess. The CT showed no liver lesions but patchy densities of the left lower lobe of lung compatible with inflammatory or infectious etiology. The erythrocyte sedimentation rate was elevated but antinuclear antibody was negative. Given lack of clear fever source, he received 10-day course of oral levofloxacin based on CT findings. His fever persisted despite antibiotic therapy thus fulfilling the FUO criteria. Preoperative staging PET/CT scan revealed a decrease in the standardized uptake value (SUV) of the GEJ mass from 28 to 13 and resolution of regional LAD indicating good local response to neoadjuvant chemoradiotherapy (Figure 1 C,D). In addition, previously noted patchy left lower lobe densities had resolved on PET/CT imaging. However, the PET/CT indicated development of a solitary 4.1 cm x 4.6 cm left hepatic lobe lesion with an SUV of 8.2 compatible with metastasis (Figure 1 D). However, since the CT scan obtained two weeks ago had not shown any hepatic lesion and patient had ongoing fever, this raised concern for an underlying hepatic abscess vs. radiation necrosis rather than metastasis. Urgent ultrasound-guided aspiration and core biopsies of the lesion were obtained. The cytology was negative for tumor cells and pathology showed hepatic venous outflow obstruction consistent with mass effect. Given clinical, radiographic and pathologic findings as well as negative infectious work-up, the fever was attributed to radiation necrosis of liver. He was started on oral prednisone 40 mg daily with dramatic improvement of fever. Prednisone was tapered over two weeks with complete resolution of fever. He subsequently underwent minimally invasive Ivor Lewis esophagectomy with good recovery.

## Discussion

An estimated 572,034 esophageal cancer cases and 508,585 deaths occured globally in 2018 [4]. In the United States alone, 17,290 esophageal cancer cases and 15,850 deaths are projected [5]. Esophageal cancer is three to four times more prevalent in males than females. There are two main histologic subtypes: squamous cell carcinoma (SCC) and adenocarcinoma. Interestingly, the incidence of SCC is decreasing in North America and Europe owing to reduced tobacco and alcohol consumption [6]. On the contrary, the incidence of adenocarcinoma is rising in the Western countries due to increased prevalence of Gastroesophageal reflux disease (GERD), Barrett’s esophagus, and obesity [6]. Almost 40% of patients have distant metastasis at diagnosis whereas ~50% have resectable disease [5]. On average, 19% of patients survive five years after diagnosis, however, five-year survival rate precipitously declines to 5% in patients with distant disease [5]. While esophagectomy remains the cornerstone treatment of clinically localized esophageal carcinoma, the nature of the disease attributes to the failure of surgery alone. In SCCs of the esophagus, neoadjuvant cisplatin was not found to offer an improvement in survival [7], while neoadjuvant cisplatin and fluorouracil increased five-year disease-free survival by 10% [8]. In contrast, neoadjuvant chemotherapy (carboplatin and paclitaxel) and radiotherapy in adenocarcinomas of the esophagus had a median overall survival of 49.4 months, compared with 24 months in the surgery-alone group [3]. Other studies have also found beneficial effects of neoadjuvant chemotherapy with epirubicin, 5-fluorouracil, and/or cisplatin in patients with adenocarcinoma of the stomach or GEJ [9-11]. On the whole, this indicates survival advantage of neoadjuvant chemoradiotherapy for adenocarcinomas of distal esophagus and GEJ. In this context, prompt recognition of neoadjuvant chemoradiotherapy complications and effective management can avoid premature termination of treatment and a chance at curative resection. Here, we present a previously unreported complication of prolonged fever due to radiation necrosis of the left hepatic lobe.

Classic RILD, which affects patients with normal pre-treatment livers, can occur through both direct treatment and also indirectly by treating a nearby disease site, such as in our case. RILD typically occurs in a timeframe from two weeks to three months after completing radiation therapy. The mechanism for RILD involves the destruction and disruption of the hepatic vasculature, namely veno-occlusive disease, leading to necrosis. Clinical symptoms generally include increase in ascites, alkaline phosphatase, and weight gain. The presence of fever is not a classic hallmark of RILD. Treatment of RILD is typically supportive in nature that includes use of diuretics for fluid retention, pain control, paracentesis to relieve ascites, and corticosteroids to prevent hepatic congestion. Constraints to mitigate such toxicity have been attempted and vary between the radiation techniques and reverse transcription (RT) protocols. Historically, for conventional fractionation and palliative whole liver radiation, mean liver dose should be limited to 30 Gy, in 2 Gy/fraction with a 5% risk of RILD in five years [12]. For partial liver, mean normal liver dose should be limited to <28 Gy, in 2 Gy/fraction for primary liver cancer and <32 Gy, in 2 Gy/fraction for liver metastases [13].

**Figure 3:**
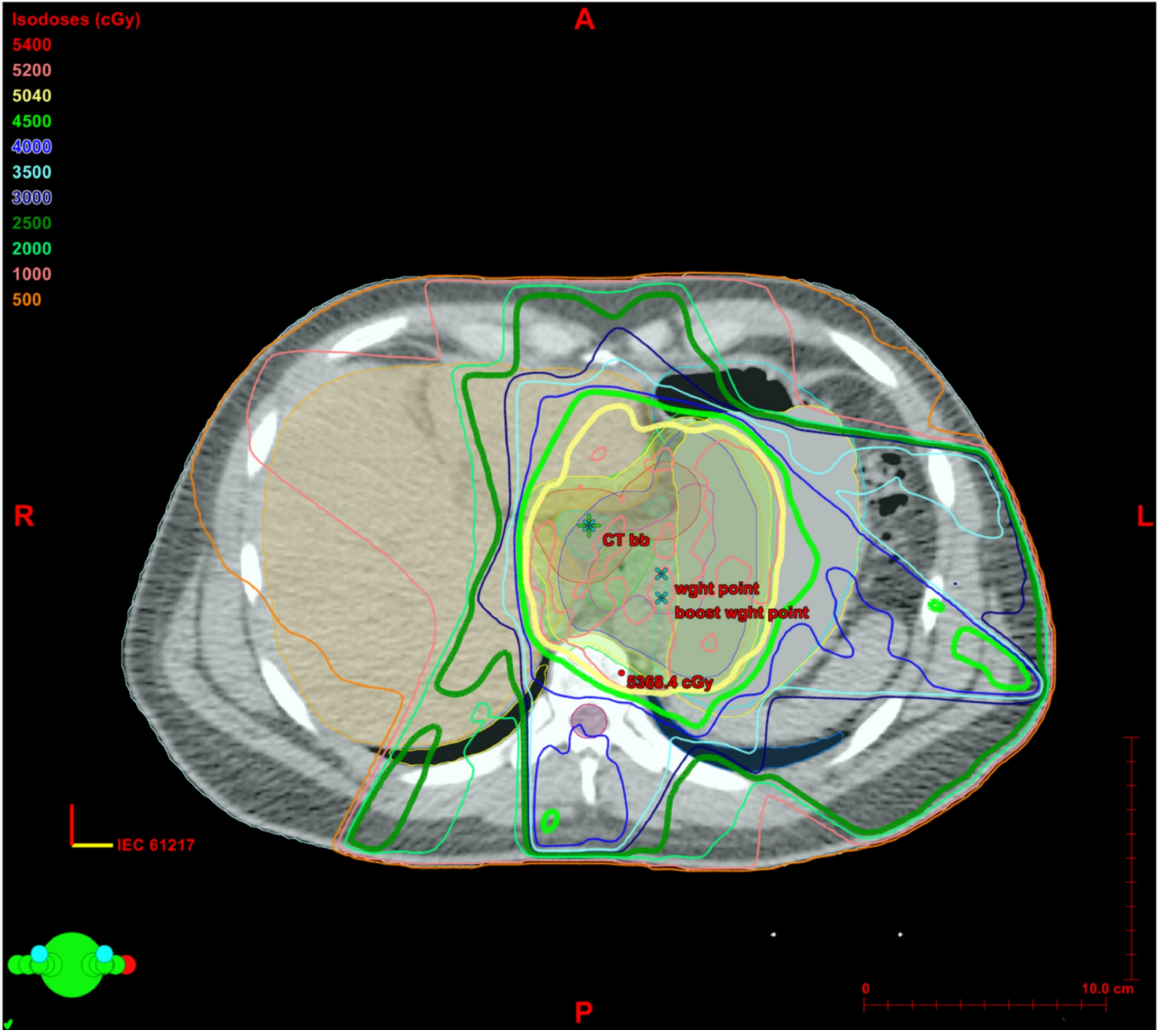
Isodose lines of radiation treatment. Axial slice of radiation treatment plan with isodose lines representing the percentage of the prescription dose delivered to the color-coded region. The area of increased standardized uptake value on PET scan in Figure 1 corresponds to the light green (45 Gy) and yellow (50.4 Gy) isodose lines.

In our case, the patient was treated to the GEJ with a total dose of 50.4 Gy delivered in 28 fractions at 1.8 Gy/fraction. By examining the isodose lines (IDL) in the radiation treatment plan (Figure 3), we can see that the region of interest corresponding to the new hepatic lesion seen on the PET scan in Figure 1D, shows exposure to the 45 Gy (89% IDL) in light green, a smaller region going to 50.4 Gy (100% IDL) in yellow.

**Figure 4:**
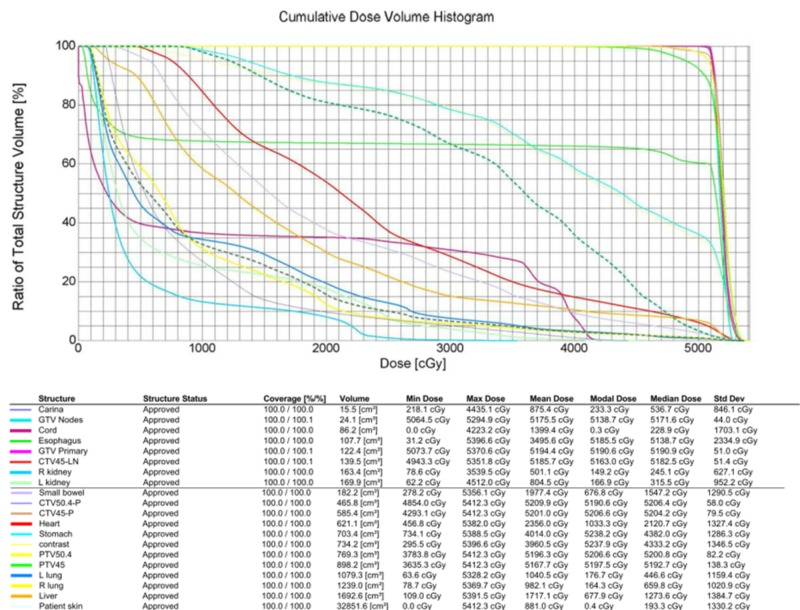
Cumulative dose volume histogram. GTV, gross tumor volume (primary and nodes); CTV, clinical target volume; PTV, planning target volume; PTV45, PTV prescribed to 45 Gy; PTV50.4, PTV prescribed to 50.4 Gy.

Our liver constraints were V30 (the volume percentage of normal liver receiving 30 Gy) < 50% and mean liver dose < 25 Gy, which our treatment plan achieved well within our goals, with a mean liver dose of 17.17 Gy and V30 of 15% as can be observed by the cumulative dose volume histogram (Figure 4).

Despite having a treatment plan meeting our dose constraints, after reviewing the IDL of radiation treatment (Figure 3) combined with the observed clinical, pathologic, and radiographic findings, it is likely that there was an area of hepatic necrosis that was induced by radiation.

A retrospective observational study previously reported that 10 of the 112 patients developed new foci of fluorodeoxyglucose avidity on PET scan of the liver during neoadjuvant chemoradiotherapy of distal esophagus, of whom nine patients were diagnosed with radiation necrosis [14]. However, prolonged fever in this setting has not been previously reported. It is important to note that there may be a lag in the appearance of radiation necrosis of liver on anatomic scans which may become apparent on functional imaging, as in our case.

## Conclusions

Fever in the absence of neutropenia, negative infectious work-up, and temporal association with radiation treatment should prompt evaluation with PET scan to rule out this unusual treatment complication. In addition, when functional imaging reveals a solitary hypermetabolic lesion of caudate or left lobe of liver after neoadjuvant chemoradiotherapy for GEJ tumor, radiation necrosis should be in the differential and confirmed with a biopsy to avoid missing the chance at potential cure.

## References

[1] Petersdorf RG, Beeson PB (1961). Fever of unexplained origin: report on 100 cases. Medicine (Baltimore).

[2] Zell JA, Chang JC (2005). Neoplastic fever: a neglected paraneoplastic syndrome. Support Care Cancer.

[3] van Hagen P, Hulshof MCCM, van Lanschot JJB (2012). Preoperative chemoradiotherapy for esophageal or junctional cancer. N Engl J Med.

[4] Bray F, Ferlay J, Soerjomataram I, Siegel RL, Torre LA, Jemal A (2018). Global cancer statistics 2018: GLOBOCAN estimates of incidence and mortality worldwide for 36 cancers in 185 countries. CA Cancer J Clin.

[5] Siegel RL, Miller KD, Jemal A (2018). Cancer statistics. CA Cancer J Clin.

[6] Torre LA, Siegel RL, Ward EM, Jemal A (2016). Global cancer incidence and mortality rates and trends—an update. Cancer Epidemiol Biomarkers Prev.

[7] Pouliquen X, Levard H, Hay JM, McGee K, Fingerhut A, Langlois-Zantin O (1996). 5-Fluorouracil and cisplatin therapy after palliative surgical resection of squamous cell carcinoma of the esophagus. A multicenter randomized trial. French Associations for Surgical Research. Ann Surg.

[8] Ando N, Iizuka T, Ide H (2003). Surgery plus chemotherapy compared with surgery alone for localized squamous cell carcinoma of the thoracic esophagus: a Japan Clinical Oncology Group Study—JCOG9204. J Clin Oncol.

[9] Macdonald JS, Smalley SR, Benedetti J (2001). Chemoradiotherapy after surgery compared with surgery alone for adenocarcinoma of the stomach or gastroesophageal junction. N Engl J Med.

[10] Ychou M, Boige V, Pignon J-P (2011). Perioperative chemotherapy compared with surgery alone for resectable gastroesophageal adenocarcinoma: an FNCLCC and FFCD multicenter phase III trial. J Clin Oncol.

[11] Cunningham D, Allum WH, Stenning SP (2006). Perioperative chemotherapy versus surgery alone for resectable gastroesophageal cancer. N Engl J Med.

[12] Emami B, Lyman J, Brown A (1991). Tolerance of normal tissue to therapeutic irradiation. Int J Radiat Oncol Biol Phys.

[13] Pan CC, Kavanagh BD, Dawson LA, Li XA, Das SK, Miften M, Ten Haken RK (2010). Radiation-associated liver injury. Int J Radiat Oncol Biol Phys.

[14] Grant MJ, Didier RA, Stevens JS, Beyder DD, Hunter JG, Thomas CR, Coakley FV (2014). Radiation-induced liver disease as a mimic of liver metastases at serial PET/CT during neoadjuvant chemoradiation of distal esophageal cancer. Abdom Imaging.

